# Nicotine-mediated invasion and migration of non-small cell lung carcinoma cells by modulating *STMN3* and *GSPT1* genes in an ID1-dependent manner

**DOI:** 10.1186/1476-4598-13-173

**Published:** 2014-07-16

**Authors:** Sajitha Nair, Namrata Bora-Singhal, Deepak Perumal, Srikumar Chellappan

**Affiliations:** 1 Department of Tumor Biology, H. Lee Moffitt Cancer Center and Research Institute, 12902 Magnolia Drive, Tampa, FL 33612, USA; 2 Department of Pediatrics, University of South Florida, 12908 Bruce B. Downs Blvd, Tampa, FL 33612, USA; 3 Department of Hematology and Medical Oncology, Icahn School of Medicine at Mount Sinai, 1 Gustave L. Levy Place, Box 1079, New York, NY 10029, USA

**Keywords:** ID1, ZBP89, NRSF, Cell proliferation, Transcriptional repression

## Abstract

**Background:**

Inhibitor of DNA binding/Differentiation 1 (ID1) is a helix loop helix transcription factor that lacks the basic DNA binding domain. Over-expression of ID1 has been correlated with a variety of human cancers; our earlier studies had shown that reported ID1 is induced by nicotine or EGF stimulation of non-small cell lung cancer (NSCLC) cells and its down regulation abrogates cell proliferation, invasion and migration. Here we made attempts to identify downstream targets of ID1 that mediate these effects.

**Methods:**

A microarray analysis was done on two different NSCLC cell lines (A549 and H1650) that were transfected with a siRNA to ID1 or a control, non-targeting siRNA. Cells were stimulated with nicotine and genes that were differentially expressed upon nicotine stimulation and ID1 depletion were analyzed to identify potential downstream targets of ID1. The prospective role of the identified genes was validated by RT-PCR. Additional functional assays were conducted to assess the role of these genes in nicotine induced proliferation, invasion and migration. Experiments were also conducted to elucidate the role of ID1, which does not bind to DNA directly, affects the expression of these genes at transcriptional level.

**Results:**

A microarray analysis showed multiple genes are affected by the depletion of ID1; we focused on two of them: Stathmin-like3 (STMN3), a microtubule destabilizing protein, and GSPT1, a protein involved in translation termination; these proteins were induced by both nicotine and EGF in an ID1 dependent fashion. Overexpression of ID1 in two different cell lines induced STMN3 and GSPT1 at the transcriptional level, while depletion of ID1 reduced their expression. STMN3 and GSPT1 were found to facilitate the proliferation, invasion and migration of NSCLC cells in response to nAChR activation. Attempts made to assess how ID1, which is a transcriptional repressor, induces these genes showed that ID1 down regulates the expression of two transcriptional co-repressors, NRSF and ZBP89, involved in the repression of these genes.

**Conclusions:**

Collectively, our data suggests that nicotine and EGF induce genes such as STMN3 and GSPT1 to promote the proliferation, invasion and migration of NSCLC, thus enhancing their tumorigenic properties. These studies thus reveal a central role for ID1 and its downstream targets in facilitating lung cancer progression.

## Introduction

Lung cancer is one of the leading causes of cancer-related deaths worldwide [1] and chemotherapy with cytotoxic agents has been the mainstay of treatment for advanced lung cancer [2]. However, the efficacy of these agents is quite limited, and they have severe adverse effects. More recently, therapies that specifically target factors involved in the development and progression of lung cancer have shown promising efficacy [3]. Greater than 85% of all lung cancer cases occur among people who are either current or former tobacco smokers [4]. Epidemiological studies clearly establish cigarette smoking as the major cause of lung cancer, as well as cancers of the pancreas, bladder and other organs [5]. It is estimated that about 90% of male lung cancer deaths and 75% of female lung cancer deaths in the United States each year are caused by smoking [6].

Nicotine, the major addictive component of cigarette smoke is not a carcinogen by itself and cannot initiate tumorigenesis in humans and rodents [7]; but many of the carcinogens in tobacco smoke are derivatives of nicotine. The addictive effects of nicotine are mediated through pentameric nicotinic acetylcholine receptors (nAChRs) that are expressed on neurons; nAChRs are also expressed on muscles, neuromuscular junctions as well as a variety of non-neuronal tissues [8]. Several studies have shown that activation of nAChRs on non-neuronal cells can induce proliferation and that nicotine has strong mitogenic effects [9,10]. Nicotine was found to induce cell proliferation in a Src dependent pathway, that was dependent on the scaffolding proteins β-arrestin-1 and E2F1 transcription factor [9]. Earlier studies had also established that nicotine could induce angiogenesis, raising the possibility that nicotine can affect the biology of vasculature; indeed, smoking has been correlated with multiple cardiovascular diseases [11]. The ability of nicotine to induce cell proliferation and angiogenesis indicated that it might have tumor promoting functions and our earlier studies showed that exposure of cells to nicotine induced epithelial-mesenchymal transition and promoted cell invasion and migration [12]. Further, nicotine was found to promote the growth and metastasis of lung and pancreatic cancers in mouse xenograft models, suggesting that nicotine can promote the growth and metastasis of tumors already initiated by tobacco carcinogens [13,14].

The two most commonly mutated oncogenes in lung cancer encode the epidermal growth factor receptor (EGFR) and K-Ras [15]. EGFR kinase domain mutations are prevalent in lung cancers in non-smokers and have been established as valid predictors of increased sensitivity to EGFR kinase inhibitors [16]. EGFR has become an important therapeutic target for the treatment of lung cancer especially in non-smokers, since more than 60% of NSCLCs express EGFR [17]. On the other hand, K-Ras mutations in lung cancer are correlated with tobacco use and K-Ras gene is known to be mutated by tobacco-specific nitrosamines. Given that K-Ras mutations and EGFR mutations give rise to lung adenocarcinomas as well as squamous cell carcinomas, we made attempts to identify any common downstream effectors of these genes. Our earlier studies showed that the ID1 gene is a downstream mediator of both K-Ras and EGFR signaling, and that ID1 can facilitate cell proliferation, invasion and migration [18]. ID1 is a member of the helix-loop-helix protein family and is expressed in a variety of tumor types [19,20]; increased expression of ID1 has been shown to be associated with decreased cell differentiation and enhanced cell proliferation, angiogenesis and metastasis of various cancers, including lung cancer [21,22]. ID1 exerts its function by acting as dominant negative transcriptional repressors of bHLH factors [23]; we found that ID1 is up regulated in response to nicotine and EGF *via* nAChR and EGFR in various lung cancer cell lines [18]. In this current paper we have identified STMN3 (Stathmin like 3) and GSPT1 (G1 to S phase transition) proteins to be major downstream targets of ID1 in NSCLC.

STMN3 is a microtubule destabilizing protein belonging to the stathmin family of phosphoproteins, along with stathmin like 2 superior cervical ganglion 10; SCG10) and stathmin-like 4 (RB3 with two splice variants, RB3′ and RB3′′). Co-expression of STMN3 and stathmin induced cell proliferation, migration, and matrix invasion in adenocarcinoma as well as squamous cell carcinoma tissues and reduced stathmin and STMN3 levels affected cell morphology and is associated with a less malignant phenotype [24]. Tumor cell growth, survival, and dissemination particularly depend on highly efficient turnover of the microtubule network which contributes to cellular processes such as cell division and migration. Several factors have been identified which facilitate dynamic microtubule instability in cancer cells, and the modulation of microtubule dynamics represents a promising therapeutic strategy.

Another protein known as GSPT1 also appears to play a major role in mediating ID1 function. Eukaryotic release factor 3(eRF3) or GSPT1 is a GTPase that associates with eRF1 in a complex mediates that translation termination. Apart from its role in the translation termination, GSPT1 has been shown to play several roles in critical cellular processes such as cell cycle regulation, cytoskeleton organization and apoptosis [25]. It has been shown recently that translation termination factors are also involved in cancer development and that components of the translation machinery that are deregulated in cancer cells. We find that GSPT1 is up regulated upon nicotine stimulation, in an ID1 dependent manner, similar to STMN3.

The results presented here show that STMN3 and GSPT1 are induced by nicotine and EGF in multiple NSCLC cell lines in an ID1 dependent manner; depletion of ID1 prevented their induction. Further, GSPT1 and STMN3 were necessary for ID1 to promote cell proliferation and invasion. We also present data that suggests ID1, which is a transcriptional repressor, induces GSPT1 and STMN3 at the transcriptional level, through the down regulation of transcriptional repressors NRSF and ZBP89. Thus, the studies presented here identify novel pathways involved in the proliferation and invasion of non-small cell lung cancer cells and opens up new avenues to combat this disease.

## Results

### STMN3 and GSPT1 are ID1 regulated genes

**Figure 1:**
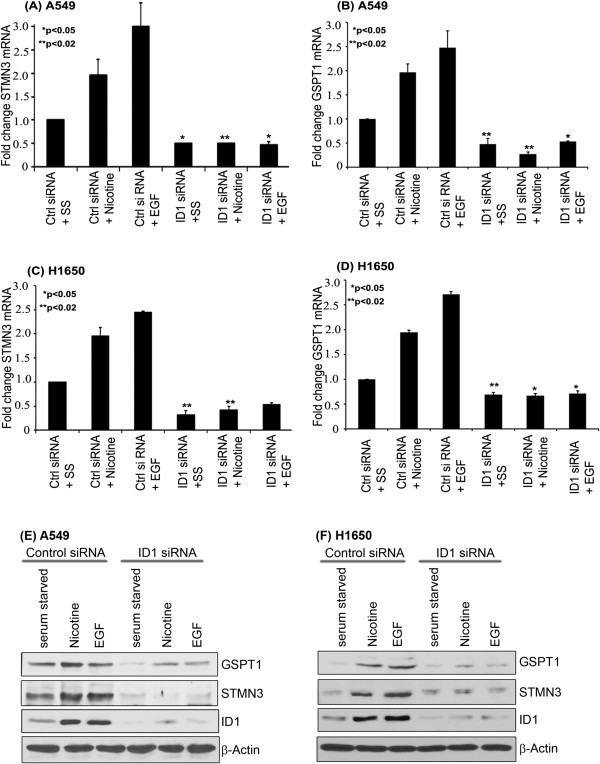
**Nicotine and EGF induces expression of STMN3 and GSPT1 in NSCLC cell lines; and depletion of ID1 by siRNA reduced this induction. (A & B)** RT-PCR showing significant fold reduction in STMN3 and GSPT1 in A549. **(C & D)** Similar results were obtained in H1650 cells, **(E)** Western blot analysis showing the upregulation of STMN3 & GSPT1 proteins by nicotine and EGF in non-targeting control siRNA cells and absence of induction in ID1 depleted A549 cells, **(F)** similar results were obtained in H1650 cells. The above data are expressed as mean ± SD of three independent experiments. *represents *p* value of <0.05; **represents *p* value of <0.02.

Previously our lab had shown that stimulation of NSCLC cells with nicotine or EGF could lead to the induction of the ID1 transcription factor, which facilitated the proliferation, invasion and migration of cells [18]. Attempts were made to elucidate the downstream molecular targets of ID1 that mediate these functional effects, since ID1 is a transcriptional repressor that does not bind to DNA. A549 cells were transiently transfected with ID1 siRNA or a non-targeting control siRNA, serum starved for 24 hours and stimulated with 1 μM nicotine or 100 ng/ml EGF for 18 hours. The serum starvation step was to reduce background signaling events mediated by growth factors present in the serum, which could mask the effects of nicotine and EGF. RNA prepared from these cells after treatment were subjected to microarray analysis. A list of genes that were up regulated or down regulated upon nicotine stimulation of A549 cells, or A549 cells depleted of ID1 is listed (Additional file 1: Tables S1 & S2). Comparing genes that were up regulated by nicotine in A549 cells but were not induced when ID1 was depleted identified multiple genes, raising the possibility that these genes are induced by nicotine in an ID1 dependent manner, and are probably mediators of ID1 function. ID1 itself was induced by nicotine as expected; in this study we focused on STMN3 and GSPT1, since they were affected by both nicotine and EGF (data not shown).RT-PCR was performed to confirm the induction of STMN3 and GSPT1 by nicotine and EGF in A549 (Figure 1A, B) and H1650 (Figure 1C, D) cells. Both STMN3 and GSPT1 were induced by 1 μM nicotine and 100 ng/ml EGF in non-targeting control siRNA transfected cells. As can be seen, the induction by both the agents was abolished when ID1 was depleted from the cells by siRNA transfection. This data validates the microarray analysis and suggests a role for ID1 in the upregulation of these genes. Additionally, the upregulation of STMN3 and GSPTI proteins in response to nicotine and EGF in A549 & H1650 cells were verified by western blotting; in agreement with the RT-PCR results, nicotine and EGF could induce STMN3 and GSPT1 proteins in both A549 (Figure 1E) and H1650 (Figure 1F) cells; there was no induction in ID1 siRNA transfected cells, showing a definite role for ID1 in mediating the induction of these genes in response to nicotine and EGF.

### STMN3 and GSPT1 are induced by Nicotine and EGF

**Figure 2:**
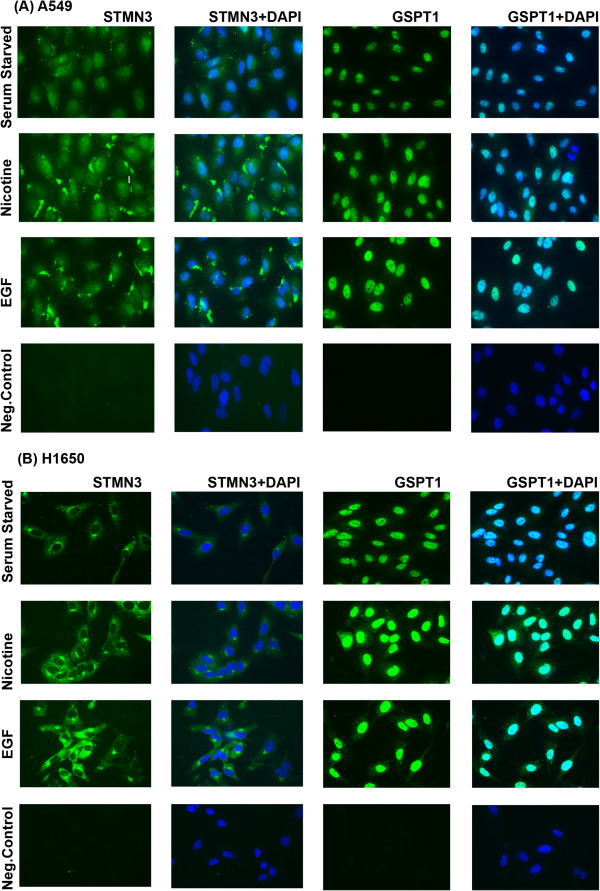
**Immunofluorescence experiments confirming the upregulation of STMN3 and GSPT1 in A549 and H1650 cells exposed to nicotine and EGF as compared to serum starved cells. (A)** A549 cells fixed and stained showing increased cytosolic expression of STMN3 and nuclear localization of GSPT1, 400X magnification **(B)** similar findings observed in the H1650 cells fixed and stained showing increased cytosolic expression of STMN3 and nuclear localization of GSPT1, 400X magnification. Three independent experiments were done and analyses were based on multiple fields.

Since RT-PCR experiments showed that the two genes were induced by nicotine and EGF at the RNA level, immunofluorescence experiments were conducted to assess whether this correlated with an increase in protein levels as well. A549 and H1650 cells were serum starved for 24 hours and stimulated with 1 μM nicotine and 100 ng/ml EGF for 24 or 48 hours and the expression of STMN3 and GSPT1 was examined by immunofluorescence. It was found that STMN3 was distributed in the cytoplasm and its levels were elevated in response to nicotine or EGF stimulation. GSPT1 showed a nuclear localization and was induced by both EGF as well as nicotine (Figure 2). The results are quantified in Additional file 1: Figure S1. This result suggests that both nicotine and EGF can induce STMN3 and GSPT1 at the protein level as well; the induction is probably a transcriptional event, given the RT-PCR results.

### Down regulation of STMN3 and GSPT1 inhibits Nicotine and EGF induced proliferation

**Figure 3:**
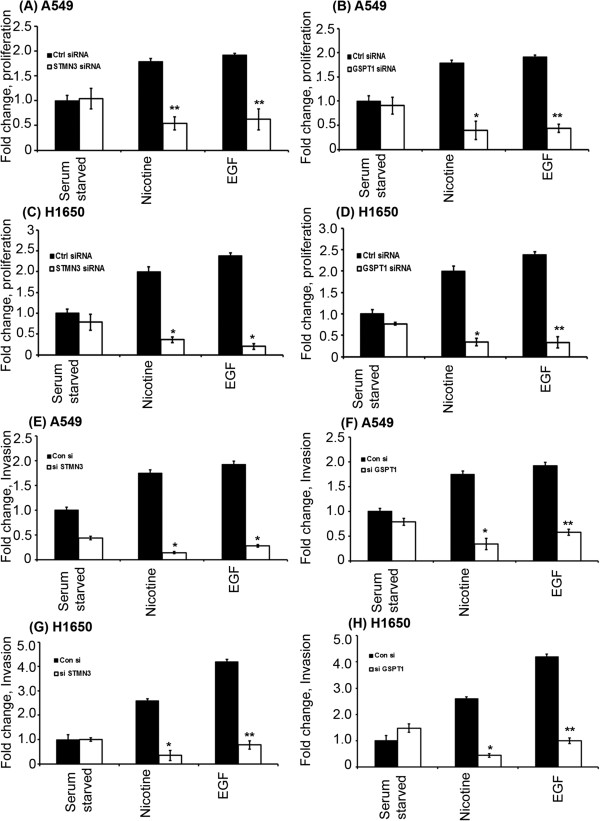
**Depletion of STMN3 and GSPT1 by siRNA transfection in NSCLC cells significantly reduces nicotine and EGF induced cell proliferation and invasion. (A & B)** showing reduced cell proliferation of A549 cells in response to nicotine and EGF, when STMN3 and GSPT1 are depleted, **(C & D)** Similar results were obtained in H1650 cells. The results are representative of three independent experiments done in duplicates. **(E & F)** Depletion of STMN3 and GSPT1 inhibits the invasion of A549 cells upon stimulation with nicotine and EGF as compared to serum starved cells, **(G & H)** similar inhibition was observed in the H1650 cells. The above data are expressed as mean ± SD of three independent experiments. *represents *p* value <0.05 and **represents *p* value < 0.0005.

Since depletion of ID1 prevented proliferation induced by nicotine or EGF [18], attempts were made to assess whether STMN3 and GSTP1 contributed this process. Towards this purpose STMN3 and GSPT1 were depleted in A549 and H1650 cells by siRNA transfection; a non-targeting siRNA was used as control. RT-PCR experiments showed that basal levels of both STMN3 and GSPT1 were down regulated by the siRNA; further, siRNA transfection abrogated their induction by nicotine or EGF in both A549 and H1650 cells (Additional file 1: Figure S2). To assess the role of these proteins in cell proliferation, the siRNA transfected cells were serum starved for 24 hours and subsequently stimulated with 1 μM nicotine or 100 ng/ml EGF for 18 hours. Stimulation of cells transfected with the non-targeting control siRNA with nicotine or EGF led to a robust incorporation of BrdU suggesting S-phase entry; in contrast, BrdU incorporation was greatly reduced in cells transfected with siRNAs to STMN3 or GSPT1 (Figure 3A-3D). This suggests that STMN3 and GSPT1 play a major role in nicotine and EGF-mediated induction of cell proliferation.

### Down regulation of STMN3 and GSPT1 abrogates the invasive capacity of cells

Our earlier studies had shown that nicotine stimulation of cells could enhance the invasive properties of cells, and could promote metastasis in mice [13]. Boyden chamber assays were conducted to assess how depletion of STMN3 and GSPT1 affected the invasive property of A549 and H1650 cells. Cells were transiently transfected with 100 pmol of a non-targeting control siRNA or siRNAs to STMN3 or GSPT1, serum starved for 24 hours, and subsequently stimulated with 1 μM nicotine or 100 ng/ml EGF for 24 hours. Boyden chamber assays showed that cells transfected with the non-targeting control siRNA invaded through the collagen and Matrigel-coated filters upon nicotine and EGF stimulation (Figure 3E-3H). In contrast, the invasion was greatly inhibited in cells transfected with STMN3 and GSPT1 siRNAs, suggesting that invasion induced by these agents requires STMN3 and GSPT1. Representative images of the Boyden Chamber assay in A549 and H1650 shows the loss of invasive activity upon STMN3 & GSPT1 depletion (Additional file 1: Figure S3).

### Down regulation of STMN3 and GSPT1 abrogates migratory ability of cells

**Figure 4:**
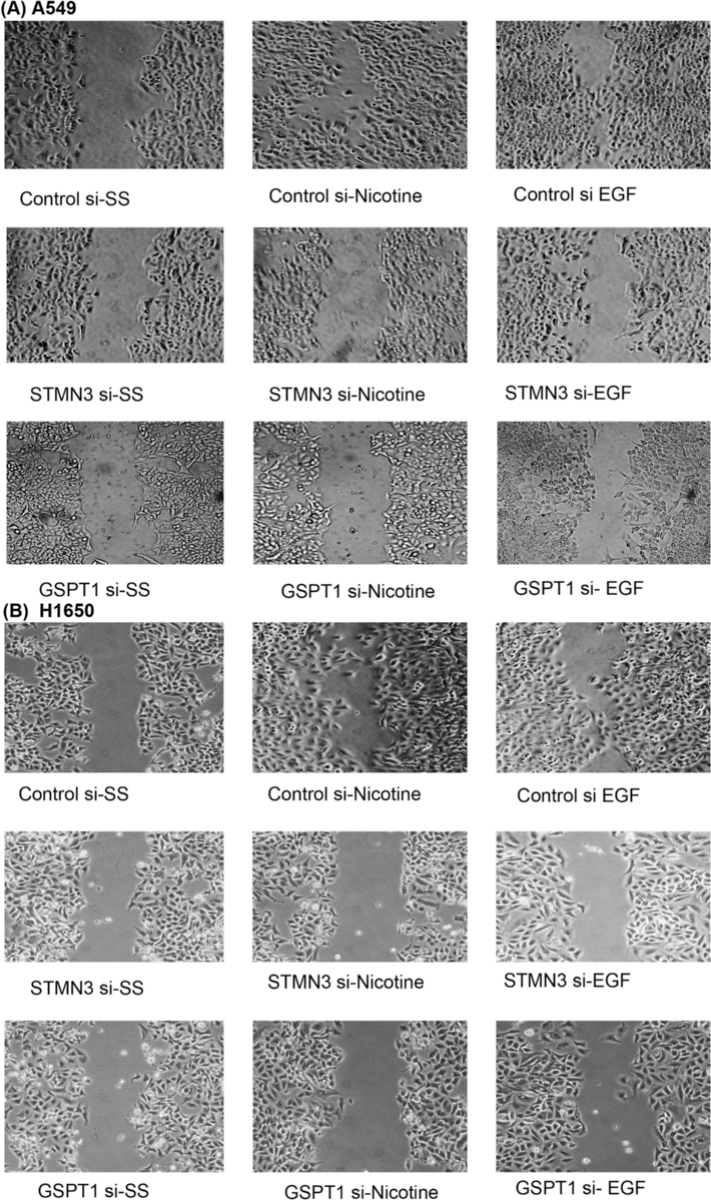
**Depletion of STMN3 and GSPT1 inhibits migration of NSCLC cells, as seen in wound-healing assays. (A)** Depletion of STMN3 and GSPT1 by transient transfection of 100 pmol of respective siRNA into A549 cells significantly reduces nicotine and EGF induced cell migration, **(B)** similar results were obtained in H1650 cells.

We next examined whether STMN3 and GSPT1 played a role in nicotine induced cell migration, using wound-healing assays. Towards this purpose, A549 and H1650 cells were transfected with siRNAs to STMN3 and GSPT1 or a non-targeting control siRNA and grown to 80-90% confluency on 35 mm dishes, and a wound-healing assay was conducted to assess cell migration in response to nicotine or EGF stimulation. It was found that non-targeting control siRNA transfected cells migrated into the wound in response to EGF or nicotine stimulation. At the same time, cells transfected with siRNAs to STMN3 or GSPT1 showed significantly reduced migration, especially in response to nicotine (Figure 4A & B). There was minimal migration in response to EGF. This result suggests that STMN3 and GSPT1 play a role in the migratory capacity of cells, in addition to proliferation and invasion.

### STMN3 and GSPT1 are elevated in cells stably over expressing ID1

**Figure 5:**
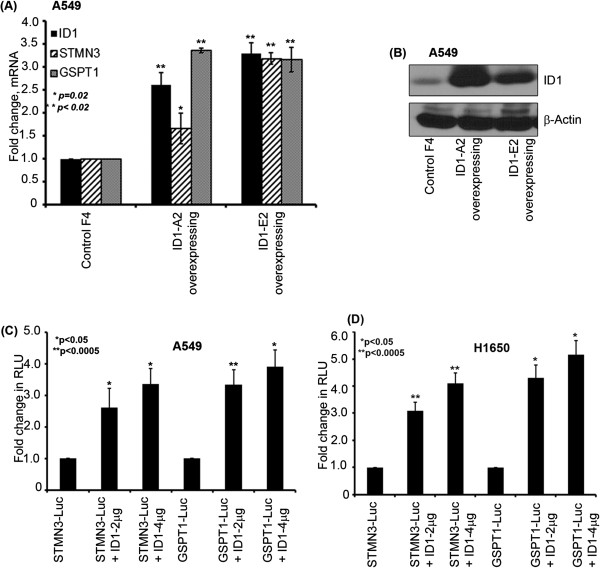
**Stable transfection confirming the ID1 overexpression in A549 cell and dose dependent induction of STMN3 & GSPT1 by ID1 and depletion of ID1 elevates the expression of STMN3 & GSPT1. (A)** The overexpressing (OE) clones showed upregulation of STMN3 and GSPT1 in ID1 over expressing clones as compared to the non-targeting control vector (pcDNA3) as observed by RT-PCR, **(B)** ID1 protein levels were also elevated as seen in a western blot in the selected clones using G418 resistance confirming the ID1 over-expression, **(C)** ID1 could induce STMN3-luciferase and GSPT1-luciferase promoters in a dose dependent manner in transient transfection experiments in A549 cells, **(D)** and H1650 cells as shown in the graph.

Since we found that depletion of ID1 could reduce the levels of STMN3 and GSPT1 messages, we examined whether over-expression of ID1 elevates these genes in two clones of A549 that stably over-expressed ID1 protein. Over-expression of ID1 was confirmed by both RT-PCR (Figure 5A) as well as western blotting (Figure 5B) experiments. Both experiments showed that levels of STMN3 and GSPT1 messages and protein were elevated in the stable clones compared to the parental cells. This experiment provides additional support to the finding that ID1 can induce STMN3 and GSPT1 in NSCLC cells.

### ID1 induces the promoters of STMN3 and GSPT1

Experiments were conducted to assess whether ID1 could induce the STMN3 and GSPT1 promoters. For this purpose, approximately 2000 bp promoter regions of STMN3 and GSPT1 were cloned in pGL3 basic vector. A549 and H1650 cells were transiently transfected with luciferase reporters driven by STMN3 and GSPT1 promoters. Co-transfection of increasing amounts of ID1 enhanced the expression of the two promoters in both the cell lines in a dose-dependent manner (Figure 5C & D). There was approximately a 3 fold increase in the luciferase activity using as low as 1 μg of STMN3 and GSPT1 promoter and 2 μg ID1. These results suggest that ID1 induces the expression of the STMN3 and GSPT1 at the transcriptional level.

### ID1 regulates STMN3 and GSPT1 expression by repressing ZBP89 and NRSF

**Figure 6:**
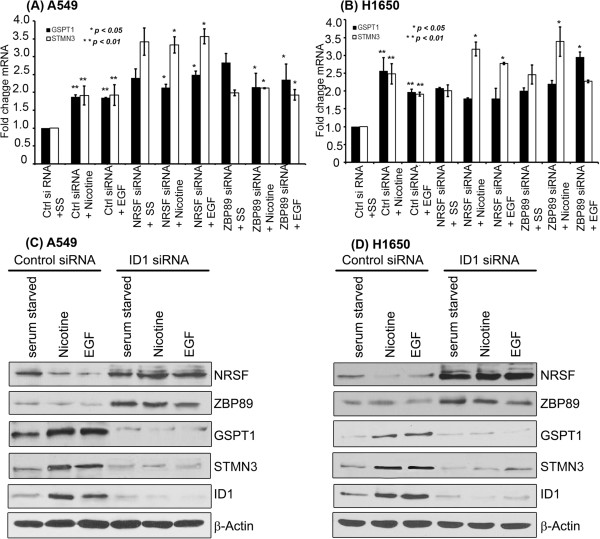
**Role of NRSF and ZBP89 in modulating the expression of STMN3 and GSPT1. (A)** Depletion of NRSF or ZBP89 leads to the induction of STMN3 and GSPT1 in A549 cells, as seen by a RT-PCR experiment. **(B)** Similar results were obtained in H1650 cells. **(C)** Western blot showing the induction of ZBP89 and NRSF upon depletion of ID1 in A549 cells; there was a corresponding decrease in the levels of STMN3 and GSPT1. **(D)** A western blot on H1650 cells showed essentially identical results.

**Figure 7:**
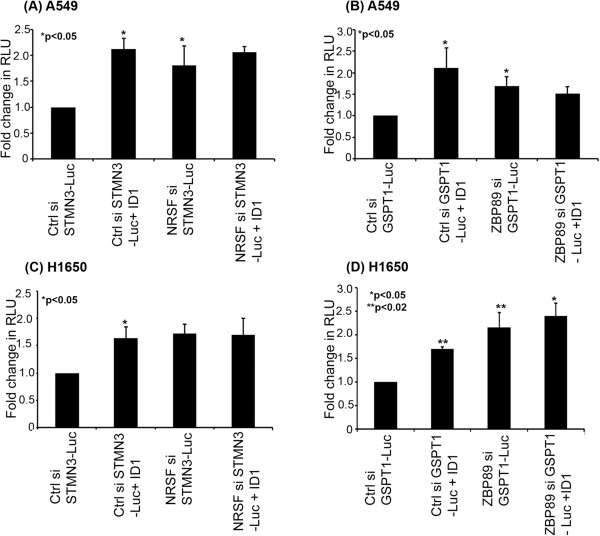
**Depletion of NRSF or ZBP89 leads to an induction of STMN3 and GSPT1. (A)** A transient transfection experiment on A549 cells showing that depletion of NRSF leads to an induction of STMN3-Luc, comparable to the induction by co-transfecting ID1; there was no further induction when ID1 was co-transfected with NRSF siRNA. **(B)** In a similar co-transfection experiment on A549 cells, depletion of ZBP89 led to an induction of GSPT1, and there was no further induction when ID1 was co-transfected. **(C)** and **(D)** show similar results that were obtained in H1650 cells. The above data are all expressed as mean ± SD of three independent experiments. *represents *p* value of <0.05; **represents *p* value of <0.02.

ID1 is an established transcriptional repressor, while and we find that it induces the transcription of STMN3 and GSPT1. Given this observation attempts were made to assess how ID1, which is a transcriptional repressor, induces gene expression. One potential mechanism is through the repression of transcriptional co-repressors. Our earlier studies had shown that ID1 could induce the mesenchymal genes fibronectin and vimentin through the mediation of the transcriptional repressor, ZBP89 [18]. Promoter analysis revealed several putative binding sites for the previously reported repressor ZBP89 on the proximal promoter of STMN3. Besides that, binding sites for another transcriptional repressor, NRSF, were also found on the promoter region of STMN3 as reported elsewhere [26]. Given this background, we performed a RT-PCR experiment to determine the role of ZBP89 and NRSF in the induction of GSPT1 and STMN3. A549 and H1650 cells were transfected with non-targeting control siRNA, ZBP89 siRNA and NRSF siRNA; it was found that depletion of ZBP89 and NRSF induced an upregulation of STMN3 and GSPT1 (Figure 6A & B). This result suggests that ID1 might be inducing STMN3 and GSPT1 by repressing ZBP89 and NRSF. Western blots were conducted on lysates from A549 cells and H1650 cells that were transfected with a control siRNA or an ID1 siRNA, and stimulated with nicotine or EGF. It was found that depletion of ID1 led to an increase in the levels of NRSF and ZBP89 (Figure 6C, D). Western blotting of the same lysates showed a corresponding decrease in STMN3 and GSPT1 levels, further supporting the notion that ID1 induces STMN3 and GSPT1 by repressing the levels of ZBP89 and NRSF. Depletion of ID1 led to an induction of ZBP89 and NRSF as seen by RT-PCR (Additional file 1: Figure S4), and suppressed cell proliferation as seen by a MTT Assay.To further confirm the role of NRSF and ZBP89 in the regulation of STMN3 and GSPT1, transient transfection experiments were conducted, using STMN3 and GSPT1 promoter-luciferase constructs in cells transfected with a non-targeting control siRNA or si RNAs to NRSF or ZBP89. When NRSF was depleted, there was an increased transcription of STMN3 promoter in A549 (Figure 7A) or H1650 (Figure 7C), while depletion of ZBP89 resulted in increased transcription of GSPT1 promoter in A549 (Figure 7B) and H1650 (Figure 7D) cells). Similarly, there was no further induction of STMN3 and GSPT1 when ID1 was co-transfected with NRSF siRNA or ZBP89 siRNA, suggesting that NRSF and ZBP89 are the main facilitators of ID1 mediated induction of STMN3 and GSPT1 expression.

## Discussion

Nicotine, the main addictive component of the cigarette smoke, can promote the growth and metastasis of lung cancers by modulating various signaling cascades. One downstream mediator of nicotine functions is the bHLH transcription factor, ID1 [18], which is known to promote oncogenesis by enhancing cell proliferation and inhibiting differentiation. The fact that both nicotine and EGF could induce the expression of ID1 in a Src-dependent manner raises the possibility that ID1 might be contributing to the genesis of lung cancers in both smokers and nonsmokers. These findings are supported by the fact that ID1 is often elevated in a variety of tumors, many of which are not correlated with smoking. Our results showing the tumor promoting functions of ID1 are also supported by a recent report showing that ID1 promotes NSCLC cell migration [21].

The finding that STMN3 gene is a downstream target of the ID1, and responds to signaling from nAChRs and EGFR in lung cancer cells is relevant for various reasons. Stathmin (STMN) is an evolutionarily conserved, ubiquitously expressed, tubulin-binding protein that has been associated with cell proliferation and differentiation. It is a member of a phosphoprotein family that also includes Stathmin-like 2 (STMN2), Stathmin-like 3 and Stathmin-like 4 (STMN4). In addition to its well-known role in cell division, STMN3 is also involved in other microtubule-dependent processes such as cell motility [27,28]. STMN3 is over-expressed in adenocarcinoma as well as squamous cell carcinoma (SCC) samples and STMN3 promoted cell proliferation, migration, and invasion [24]. In addition, reduced STMN3 level affected cell morphology and was associated with a less malignant phenotype. The substantial expression of STMN3 in most tissues point out a novel function for this protein outside the nervous system and raises the possibility that modulation of STMN3 could promote nicotine mediated induction of non-small cell lung cancer progression and metastasis. In addition to post-translational modifications, STMN3 is regulated by transcription factors such as Egr1, p53 and members of the E2F family [29]. Another study demonstrated that STMN3 is required to maintain cell–cell adhesion in MCF-7 cells and its down-regulation contributes to the loss of epithelial morphology [30]. Over-expression of stathmin family members has been reported in hepatocellular carcinoma [31], sarcoma [32], and lung adenocarcinomas [33] and poorly differentiated tumors of the breast and ovaries express higher levels of stathmin than more differentiated and less proliferative tumors [27]. Recent studies have also revealed STMN3 contributes to chromosome instability leading to aneuploidy, cell migration and chemo-resistance [34,35], which could be due to the regulation of microtubule stability by STMN3. These studies suggest that STMN3 potentially contributes to tumor development and progression through the regulation of multiple cellular processes and is probably a good target for the development of therapeutic agents.

It is now widely recognized that translation factors are involved in cancer development and that components of the translation machinery that are deregulated in cancer cells may become targets for cancer therapy. The eukaryotic Release Factor 3 (eRF3) is a GTPase that associates with eRF1 in a complex that mediates translation termination. In humans, eRF3 has two distinct isoforms, eRF3a encoded by eRF3a/GSPT1 gene and eRF3b, encoded by eRF3b/GSPT2 gene [36,37]. The N-terminal domain of GSPT1 has been shown to participate in essential protein interactions necessary for different functions such as the formation of the translation termination complex [38]. Apart from its pivotal role in translation, the N-terminal domain of GSPT1 was also reported to interact with various proteins with different biological functions and also to act as a regulator of apoptosis [39]. GSPT1 mRNA is abundant in all tissues and its level varies during the cell cycle, whereas GSPT2 mRNA is poorly expressed in most mouse tissues tested, except in brain [40]. It has been previously reported that GSPT1 mRNA level is increased in 70% of the intestinal type gastric tumors [41] and strongly decreased during human chondrocyte differentiation [42]. Understanding GSPT1 gene regulation and its relation with cell cycle progression and cellular proliferation may have prognostic value and potential therapeutic applications.

An additional interesting observation made in these studies is that the transcriptional repressors NRSF and ZBP89 are modulated by ID1 to effect the induction of STMN3 and GSPT1. NRSF, or neuron-restrictive silencer factor, is known to bind to neuron-restrictive silencer elements to repress transcription of various genes involved in neuronal functions; it has also been shown to have functions in non-neuronal cells and cancers of various organs. A role for this factor has been proposed in various neuronal disorders, including Huntington’s Disease. It is intriguing that multiple genes and transcriptional co-factors that are predominantly shown to play a role in neuronal functions are altered in NSCLC cells. It is possible that these genes as well as transcription factors respond to nAChRs, which are expressed at high levels in neurons and neuromuscular junctions. Thus, the involvement of such proteins might be indicative of a generalized function for them downstream of nAChRs, or it is possible that they also contribute to NSCLC. Earlier studies from our lab has shown that expression of vimentin and fibronectin is regulated by ID1 at transcriptional level, by down regulating ZBP89, which is a transcriptional repressor of vimentin and fibronectin [43,44]. These studies report ZBP89 regulating multiple aspects of tumor development including cell proliferation and apoptosis. Linking ZBP89 to ID1 presents an interesting scenario where a transcriptional repressor down regulates a second repressor, to promote the expression of various genes that promote cell proliferation and invasion.

As mentioned earlier, ID1’s proliferative and anti-apoptotic functions have been correlated with the onset and progression of a variety of human tumors, including those of the mammary gland and pancreas [45,46], and its over expression is significantly associated with increased tumor angiogenesis and a worse prognosis in these cancers [47]. Taken together, our studies strongly suggest that nicotine and EGF might be promoting the proliferation, invasion and migration of non-small cell lung cancer cells *via* up regulating ID1, which leads to the induction of downstream proteins like STMN3 and GSPT1 through the involvement of transcriptional repressors like ZBP89 and NRSF. These proteins might turn out to good targets for combating NSCLC.

## Conclusions

In summary, our studies show that ID1 plays a significant role in promoting cell proliferation, invasion and migration downstream of nAChRs and EGFR. These tumor-promoting functions of ID1 appear to be brought about by STMN3 and GSPT1, which are up regulated by ID1. These studies also presents an interesting scenario where ID1, which is a transcriptional repressor, induces downstream targets by repressing established transcriptional repressors like NRSF and ZBP89. Our results raise the possibility that alterations in these gene regulatory pathways lead to the genesis and progression of NSCLC and targeting these regulatory molecules might be a viable strategy to combat NSCLC.

## Materials and methods

### Cell lines and reagents

The two human NSCLC cell lines used in this study were A549 (K-Ras mutant) and H1650 (EGFR mutant) obtained from ATCC. These cell lines were maintained in Ham’s F-12 K and RPMI-1640 (Mediatech Cellgro, USA) supplemented with 10% FBS (Mediatech Cellgro, USA). Nicotine (Sigma, USA) and EGF (Sigma, USA) used in the studies were 1 μM and 100 ng/ml respectively for 18–24 hours depending on the experiment. The cells were grown to 70% confluency in complete medium with serum for 24 hours and rendered quiescent by serum starvation for 48 hours and stimulated with nicotine and EGF for 18–24 hours for analysis.

### Microarray data analysis

A549 and H1650 cells were transfected with a siRNA to ID1 or a control, non-targeting siRNA, subjected to serum starvation for 48 hours and subsequently stimulated with nicotine for 18 hours. Total RNA extracted from the samples were used to generate cDNA targets, which were hybridized to Human Genome U133A plus 2.0 oligonucleotide probe arrays (Affymetrix, Santa Clara, CA) according to standard protocols. Raw data was processed by log2 transformation of the expression values, and the mean center expression level for each gene was determined. We looked for genes that were over- or under expressed upon ID1 depletion, whose expression was altered at least two fold. Genes that were differentially expressed upon nicotine stimulation and ID1 depletion were analyzed. The data discussed in this publication has been deposited in NCBI’s Gene Expression Omnibus through GEO Series accession number GSE38944.

### siRNA transfections and real time PCR

The siRNA oligonucleotides for ID1 (SC-29356), STMN3 (SC-76459) and GSPT1 (SC-93210) were purchased from Santa Cruz Biotechnology. A non-targeting siRNA was used as the control. A549 and H1650 cells were grown in 60 mm dishes and transfected either with control siRNA (100 pmol) or target siRNA (100 pmol) using Oligofectamine (Invitrogen, CA) in as per the manufacturer’s recommendations. The total RNA was isolated using the RNeasy kit (Qiagen, CA). Levels of ID1 mRNA, STMN3 mRNA and GSPT1 mRNA were analyzed by quantitative reverse transcription PCR performed on a Bio-Rad iCycler. Data were normalized to GAPDH RNA, and fold change was represented as 2^-∆∆ct^. The primers designed for qRT-PCR used for amplifying ID1, STMN3 and GSPT1 are shown (Additional file 1: Table S3).

### Generation of stable cell lines

A549 cells that stably overexpress ID1 were generated by transfecting A549 cells with the ID1 expression plasmid construct in pcDNA3 and selecting for G418 resistance. A549 cells transfected with empty vector (pcDNA3) were used as control.

### Lysate preparation and Western blots

Lysates from A549 & H1650 cells treated with different agents and transfected (transient or stable) as per the experimental needs were subjected to NP-40 lysis method. Lysates from these cells were prepared by NP-40 lysis as described earlier and 100 μg protein was run on a polyacrylamide SDS gel. The proteins were transferred to a nitrocellulose membrane and immunoblotted with antibodies raised against various proteins. Monoclonal ID1 antibody was purchased from Biocheck, USA (BCH-1; Cat .no# 195–14), Monoclonal NRSF and ZBP89 antibody (Cat.no# SC-374611 & Cat.no# SC-137171), polyclonal STMN3 (Cat.no# SC-85907) from Santa Cruz Biotechnology, polyclonal GSPT1 from proteintech (Cat.no: 10763-1-AP) and monoclonal antibody to actin was purchased from Sigma, USA.

### Cell proliferation assays

A549 and H1650 cells were plated on poly-D-lysine coated glass chamber slides at a density of 5,000 cells per well and transiently transfected with siRNA for ID1, STMN3 and GSPT1, or a non-targeting control siRNA (100 pmol) using Oligofectamine reagent (Invitrogen, USA). Cells were serum starved for 24 hours after transfection and stimulated with 1 μM nicotine or 100 ng/ml EGF for 18 hours. Cell proliferation was assessed by BrdU incorporation assays using a kit from Roche Biochemicals, USA. BrdU incorporation was visualized by microscopy and quantitated by counting 5 fields of 100 cells in triplicate. Data presented is representative of two independent experiments and presented as the fold change of BrdU positive cells.

Cell proliferation assay was also measured with MTT (Thiazolyl Blue Tetrazolium Bromide) after 48 hours of nicotine treatment. Briefly, cells were plated in 96-well plates at a density of 7500 cells/well in triplicates. After nicotine treatment as mentioned above, they were incubated with the 1 mg/mL MTT solution at 37°C for 1 hour. The reaction was terminated with DMSO that solubilizes the formazan product formed. Absorbance at 590 nm was recorded using plate reader.

### Invasion assays

The invasion of A549 and H1650 cells was measured using Boyden Chamber assays, as described before [18]. Both the cell lines were subjected to transient transfection using ID1 siRNA, STMN3 siRNA and GSPT1 siRNA. After 24 hours of transfection, cells were rendered quiescent by serum starvation, and then treated with 1 μM nicotine or 100 ng/ml EGF for 24 hours. Briefly, the upper surfaces of the transwell filters were precoated with collagen (100 μg/filter). Matrigel was applied to the upper surface of the filters (50 μg/filter) and dried in a hood. These filters were placed in Boyden chambers (Costar, USA). Following treatment with nicotine and EGF for 24 hours, cells were trypsinized and 20,000 cells were plated in the upper chamber of the filter in media containing 0.1% bovine serum albumin (Sigma, USA) and Nicotine or EGF. Media containing 20% fetal bovine serum was placed in the lower well as an attractant and the chambers were incubated at 37°C for 18 hours. The filters were processed first by fixing them in methanol followed by staining them with hematoxylin. The cells migrating on the other side of the filters were quantitated by counting six different fields in three independent experiments under 20X magnification.

### Wound healing assay

A549 and H1650 transfected with siRNAs to ID1, STMN3 and GSPT1 were grown in a 6-well plate (Falcon Becton Dickinson, USA). These cells were grown in serum free media for 24 hours and then washed with 1x Dulbecco’s Phosphate-buffered saline (MediaTech, USA). The cells were scratched with a sterilized 200 μl pipette tip in three separate places in each well and medium containing 1 μM nicotine, EGF (100 ng/ml) or starving media was added to the wells. After 24 hours, the wounds were examined for closure by microscopy and images were taken at 20X magnification.

### Cloning of STMN3 and GSPT1 promoters

STMN3 and GSPT1 gene sequence was searched using NCBI Genome database, promoter sequence analysis was conducted using Ensembl database, the homology of the cloned STMN3 promoter sequence was confirmed using NCBI BLAST [48] software and the transcription factor binding sites were analyzed using MatInspector, Genomatix software (Genomatix Software GmbH, Munich, Germany).

Primers were designed to amplify about 2 kb upstream from the ATG region of the STMN3 and GSPT1 promoters. The PCR primers used were STMN3: forward, 5'- GAC AGA GTC TTG CTG TTT CGC C -3'; reverse, 5'- GAA CTG TCT GTG TGT GTC CTG C -3'); and GSPT1 forward,5’- GTG GGT GGG TGG GGA GTG AAA AT-3’; reverse, 5'-GCA GTG TGG CTC ATA AAG CGC TG-3’ based on the sequence retrieved from the database of Ensembl. The amplified PCR product was first cloned into pCR 2.1 TA cloning vector (Invitrogen) and subsequently sub cloned into pGL3 basic vector (Promega, USA) to generate the 2 kb reporter constructs.

### Transfections and luciferase assays

STMN3 promoter-luciferase and GSPT1 promoter-luciferase constructs were transfected into A549 and H1650 cells using FuGENE HD (Roche Diagnostics, USA). In brief cells were plated for 24 hours before transfection at a density of 85,000 cells per well and then transfected with reporter and expression plasmids as indicated; ID1 a renilla luciferase vector was used as internal control. After 48 hours, the cells were harvested and luciferase activities were determined using a microplate luminometer (Turner luminometer, USA). For each construct, relative luciferase activity was defined as the mean value of the firefly luciferase/ Renilla luciferase ratios obtained from at least three independent experiments. Dual luciferase assay system (Promega, USA) following the manufacturer’s protocol and luciferase activity was measured with a luminometer.

### Immunofluorescence

Cells grown on coverslips were washed with PBS, fixed with 10% buffered formaldehyde in PBS buffer for 15 min at RT, washed three times with PBS (50 mM Tris, 138 mM NaCl, 2.7 mM KCl, pH 7.6), permeabilized with Triton X-100 (0.25% v/v in PBS) for 5 min, before being washed twice and blocked for 1 hour in blocking solution (PBS containing 10% goat serum and 0.1% Triton X-100). Coverslips were then, incubated with antibodies anti-STMN3 (1:200) and anti-GSPT1 (1:200) (ProteinTech, USA) in a solution of 10% goat serum at RT in a humidified chamber overnight. After washing three times with PBS, slides were incubated with Alexa Fluor 488 goat anti-mouse-IgG (1:200, Invitrogen GmbH, Karlsruhe, Germany) in blocking solution for 1 hour. Slides were then washed again three times before being counterstained with DAPI (0.2 μg/ml in water) for 5 min, briefly washed with PBS, covered with anti-fade mounting medium (Vectashield, Germany) and placed onto microscope slides. Slides were examined under a Zeiss Axiovert fluorescence microscope (Carl Zeiss AG, Germany). Negative controls were performed omitting the primary antibodies. These experiments were replicated three times and the quantification of the immunofluorescence is shown in the supplementary figures (Additional file 1: Figure S3).

### Statistical analysis

Data were presented as the mean ± SD (standard deviation) from three independent experiments except where indicated. To assess the statistical significance of differences, student *t*-test and of *p* < 0.05 was considered significant.

## Abbreviations

bHLH: Basic helix loop helix; EMT: Epithelial mesenchymal transition; NRSF: Neuron –restrictive silencer factor; ZBP89: Zinc binding protein; EGFR: Epidermal growth factor receptor.

## Competing interests

The authors declare that they have no competing interests.

## Authors’ contributions

SC directed the overall study. SN carried out the functional assays with A549 & H1650 cell lines, performed quantitative RT-PCR, Immunofluorescence, generation of ID1 overexpressing stable clones, transient and stable transfections for the promoter –luciferase reporter assay experiments, cloning and construction of STMN3 promoter for the reporter assays. DP designed primers, cloning and construction of GSPT1 promoter for the reporter assays and performed transient transfections followed by luciferase assays, NBS performed the western blot analysis confirming the upregulation of STMN, GSPT1 & ID1 with response to Nicotine & EGF and downregulation of the same in ID1 depleted cells, MTT & BrdU assays provided in the supplementary data, SN, NBS & DP contributed to writing the manuscript, SC corrected and revised the manuscript, SN, DP, NBS & SC read and approved the final manuscript.

## Supplementary Material

Additional file 1: Table S1 Partial list of up regulated and down regulated genes in A549 cells treated with Nicotine. Genes that were up regulated or down regulated two fold in quiescent A549 upon stimulation with 1 μM nicotine for 18 hrs. **Table S2.** Partial list of the genes downregulated in A549 upon nicotine stimulation when ID1 expression was depleted. Genes that were down regulated two fold or more in quiescent A549 transfected with 100 pmoles of ID1 siRNA and stimulated with 1 μM nicotine for 18 hrs. **Figure S1.** Quantification of immunofluorescence in A549 & H1650 cells showing the induction of STMN3 and GSPT1 in response to Nicotine & EGF using integrated density as the parameter (supporting data for Figure 2). **Figure S2.** A549 and H1650 cells transfected with STMN3 and GSPT1 siRNA. (A & B) Transient transfection in A549 or H1650 cells (C, D) using siRNAs shows significant down regulation of the STMN3 and GSPT1 mRNA. Data expressed as mean ± SD of three independent experiments. **Figure S3.** Depletion of STMN3 and GSPT1 reduces cell invasion *in vitro* in A549 and H1650 cells. (A) Depletion of STMN3 and GSPT1 significantly inhibited the invasion induced by nicotine and EGF in a Boyden-chamber invasion assay. Cells were fixed and stained with hematoxylin and quantified as in Figure 3E-3H. **Figure S4.** Depletion of ID1 by transient transfection up regulates ZBP89 & NRSF, whereas it abrogates the cell growth & proliferation in A549 & H1650. RT-PCR showing upregulation of ZBP89 (A), NRSF (B) in the cells depleted of ID1 (C). (D) Depletion of ID1 in A549 and H1650 cells significantly reduces nicotine & EGF induced cell proliferation as seen in BrdU incorporation and viability as seen in MTT assays (E). *represents *p* value <0.05 and **represents *p* value < 0.0005. Click here for file
