# Supplementary material for: Nicotine-mediated invasion and migration of non-small cell lung carcinoma cells by modulating STMN3 and GSPT1 genes in an ID1-dependent manner
Source: Mol Cancer. 2014 Jul 16;13:173. doi: 10.1186/1476-4598-13-173 (PMC4121302; doi:10.1186/1476-4598-13-173)
Supplement: Additional file 1: Table S1 — Partial list of up regulated and down regulated genes in A549 cells treated with Nicotine. Genes that were up regulated or down regulated two fold in quiescent A549 upon stimulation with 1 μM nicotine for 18 hrs. Table S2. Partial list of the genes downregulated in A549 upon nicotine stimulation when ID1 expression was depleted. Genes that were down regulated two fold or more in quiescent A549 transfected with 100 pmoles of ID1 siRNA and stimulated with 1 μM nicotine for 18 hrs. Figure S1. Quantification of immunofluorescence in A549 & H1650 cells showing the induction of STMN3 and GSPT1 in response to Nicotine & EGF using integrated density as the parameter (supporting data for Figure 2). Figure S2. A549 and H1650 cells transfected with STMN3 and GSPT1 siRNA. (A & B) Transient transfection in A549 or H1650 cells (C, D) using siRNAs shows significant down regulation of the STMN3 and GSPT1 mRNA. Data expressed as mean ± SD of three independent experiments. Figure S3. Depletion of STMN3 and GSPT1 reduces cell invasion in vitro in A549 and H1650 cells. (A) Depletion of STMN3 and GSPT1 significantly inhibited the invasion induced by nicotine and EGF in a Boyden-chamber invasion assay. Cells were fixed and stained with hematoxylin and quantified as in Figure 3E-3H. Figure S4. Depletion of ID1 by transient transfection up regulates ZBP89 & NRSF, whereas it abrogates the cell growth & proliferation in A549 & H1650. RT-PCR showing upregulation of ZBP89 (A), NRSF (B) in the cells depleted of ID1 (C). (D) Depletion of ID1 in A549 and H1650 cells significantly reduces nicotine & EGF induced cell proliferation as seen in BrdU incorporation and viability as seen in MTT assays (E). *represents p value <0.05 and **represents p value < 0.0005. [file 1476-4598-13-173-S1.docx]

**SUPPLEMENTARY MATERIALS**

**Table 1: Partial list of up regulated and down regulated genes in A549 cells treated with Nicotine.** Genes that were up regulated or down regulated two fold in quiescent A549 upon stimulation with 1 µM nicotine for 18hrs.

| Genes | Fold change upregulated | Genes | Fold change  Downregulated |
| --- | --- | --- | --- |
| RASD1 | 3.74 | EPHB2 | -2.0118 |
| SOX8 | 2.90 | AGTR1 | -2.01235 |
| **GSPT1** | **2.77** | TRAF3 | -2.02594 |
| MKLN1 | 2.76 | CD47 | -2.04756 |
| GPR107 | 2.65 | PTPN13 | -2.04757 |
| ANXA10 | 2.60 | NRCAM | -2.05697 |
| **STMN3** | 2.54 | G0S2 | -2.06048 |
| TPD52 | 2.51 | IL8 | -2.09699 |
| VAMP3 | 2.40 | CDC42EP3 | -2.11009 |
| TFF1 | 2.33 | JUN | -2.16948 |
| **ID1** | 2.26 | CXCL3 | -2.17038 |
| FBLN1 | 2.26 | CDH2 | -2.18928 |
| EIF2S2 | 2.22 | CDKN1A | -2.20207 |
| FAM125A | 2.21 | ANXA8 | -2.21096 |
| GSPT1 | 2.20 | MMP7 | -2.21608 |
| GPD2 | 2.18 | RAB39B | -2.25765 |
| PRKCD | 2.18 | TFPI2 | -2.26455 |
| MYADM | 2.15 | RBBP6 | -2.27328 |
| HIPK3 | 2.13 | ETS1 | -2.27802 |
| PDCD4 | 2.10 | PPM1K | -2.28381 |
| NEDD1 | 2.10 | MKNK2 | -2.31394 |
| MAP4K2 | 2.10 | TNFRSF11B | -2.33801 |
| CTTN | 2.04 | RUNX2 | -2.34165 |

**Table 2: Partial list of the genes downregulated in A549 upon nicotine stimulation when the ID1 expression was depleted.** Genes that were down regulated two fold or more in quiescent A549 transfected with 100 pmoles of ID1 siRNA and stimulated with 1 µM nicotine for 18hrs.

| Genes | Fold change | Genes | Fold change |
| --- | --- | --- | --- |
| ABCG2 | -2.00 | PAK2 | -2.36 |
| **STMN3** | -2.01 | ENO3 | -2.38 |
| CTNND1 | -2.01 | ITFG2 | -2.39 |
| RAB1B | -2.04 | NEDD1 | -2.40 |
| PRKCA | -2.08 | MUM1 | -2.47 |
| RAB37 | -2.09 | MAP3K12 | -2.57 |
| ID2 | -2.09 | CCDC34 | -2.59 |
| TAF13 | -2.10 | RASD1 | -2.59 |
| RAB4B | -2.11 | CTNNA1 | -2.60 |
| GADD45B | -2.11 | **ID1** | -2.77 |
| ARHGAP5 | -2.13 | **GSPT1** | -2.79 |
| GPD2 | -2.19 | HIP2 | -2.91 |
| HIG2 | -2.20 | AXL | -2.98 |
| PAK4 | -2.23 | GSPT1 | -3.04 |
| PAK2 | -2.28 | NEDD1 | -3.23 |
| SOX8 | -2.30 | TPD52 | -3.54 |
| MAP3K12 | -2.31 | RGS20 | -4.03 |
| ZNRF2 | -2.33 | GADD45B | -4.06 |

**Table 3: qRT-PCR primers used in this study**

| PCR primers | Forward(5’) | Reverse(5’) |
| --- | --- | --- |
| ID1 | GAG CTG AAC TCG GAA TCC GAA G | GAT CGT CCG CAG GAA CGC ATG C |
| STMN3 | TGCTGTCGCTCATCTGCTCC | TCACCTCCATGTCCCCGTACT |
| GSPT1 | CGCCAGGTGCTCCTAAGAAAG | CAAATACATTATTTGTCCTCCAATGGT |
| ZBP89 | GGACTAATAGTTGGTGATTCACAGCA | GGAGCTCATTTGGTAAGCTTGGTG |
| NRSF | GTGAGCGAGTATCACTGGAGG | CCCATTGTGAACCTGTCTTGC |

**
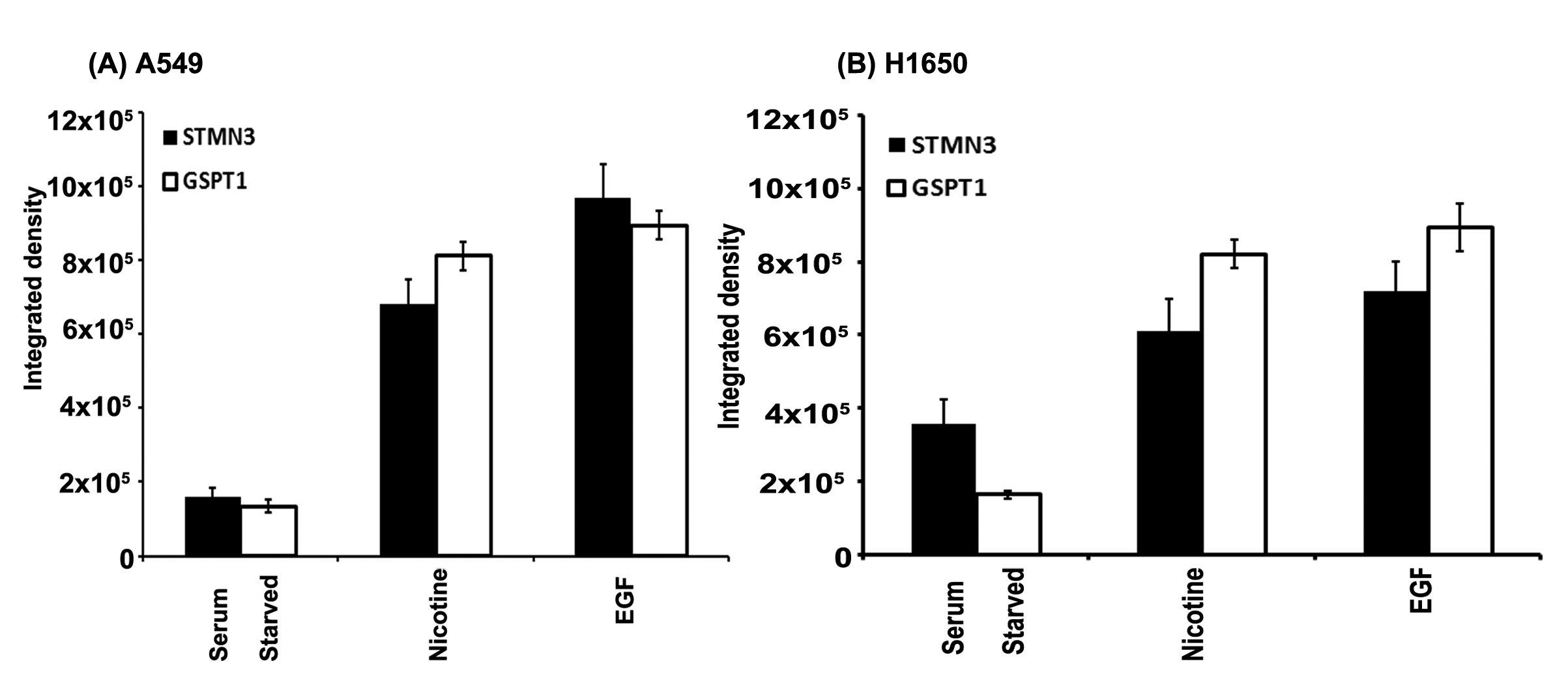
**

**Supplementary Figure S1. Quantification of immunofluorescence in A549 & H1650 cells showing the induction of STMN3 and GSPT1 in response to Nicotine & EGF using integrated density as the parameter (supporting data for Fgure 2).**

**
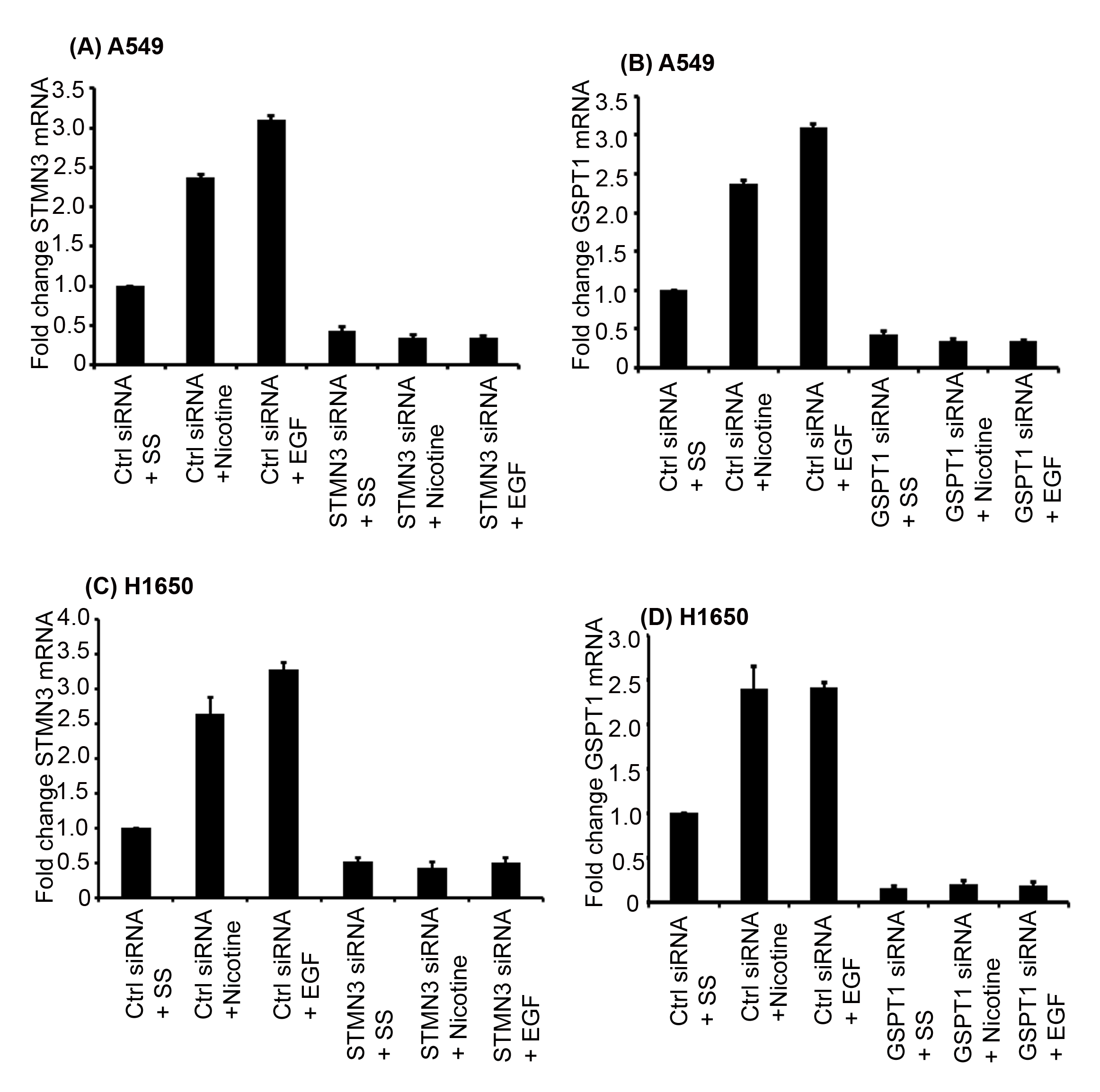
 Supplementary Figure S2:** **A549 and H1650 cells transfected with STMN3 and GSPT1 siRNA.** (A & B) Transient transfection in A549 using the siRNA shows significant down regulation of the STMN3 and GSPT1 mRNA compared with the non-targeting control siRNA as seen by RT-PCR, (C & D) Similar down regulation of STMN3 and GSPT1 was observed in H1650 cells by RT-PCR. The above data are all expressed as mean ± SD of three independent experiments.

**
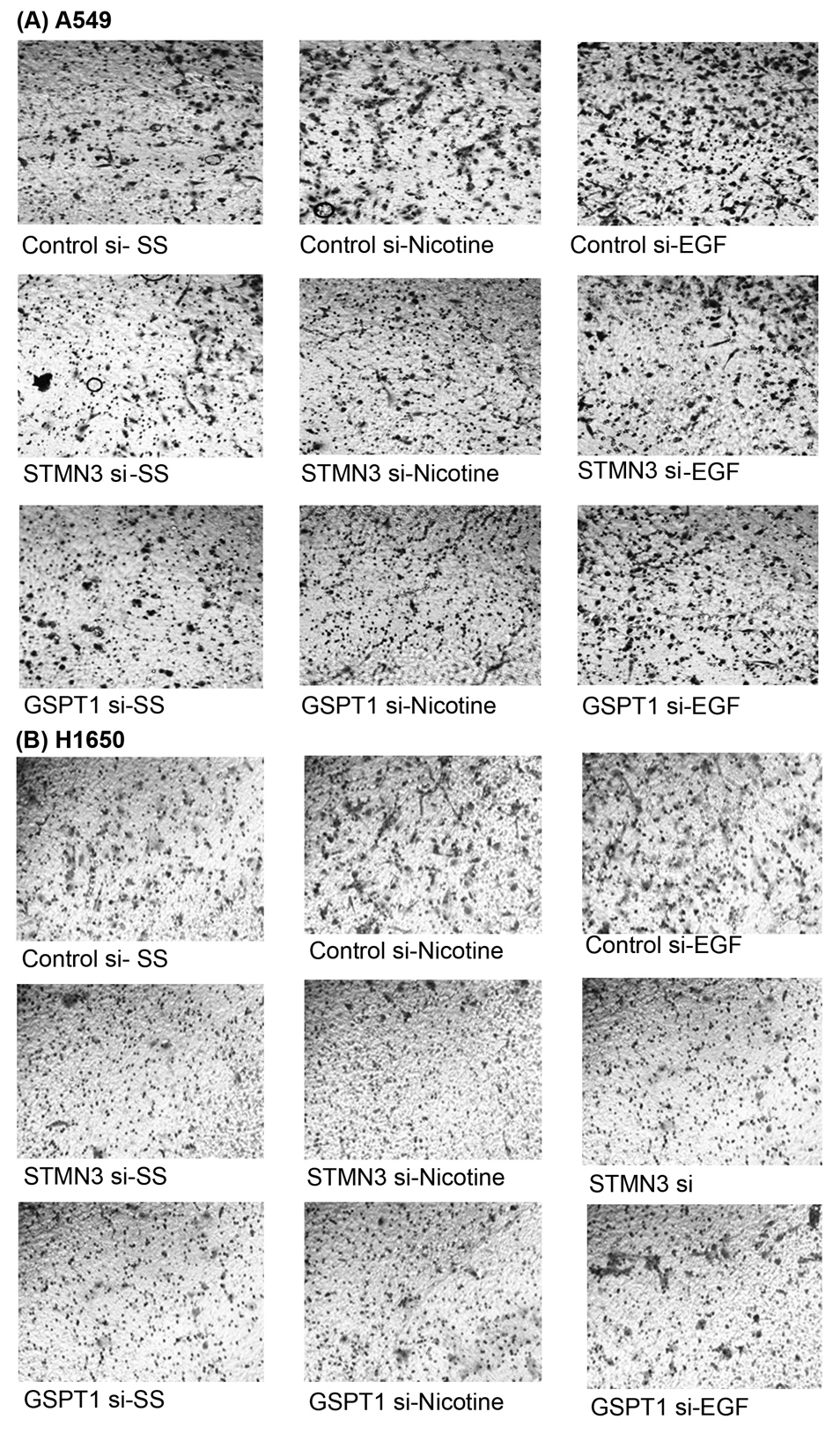
**

**Supplementary Figure S3:** **Depletion of STMN3 and GSPT1 reduces cell invasion *in vitro* in A549 and H1650 cells.** (A) Representative images of A549 cells that invaded through the matrigel in transwell invasion assay. Depletion of STMN3 and GSPT1 significantly inhibited the invasion as compared to the non-targeting control siRNA in presence of nicotine and EGF. Cells were ﬁxed and stained with hematoxylin. Cells were quantiﬁed as shown in Figure 3E-3H.


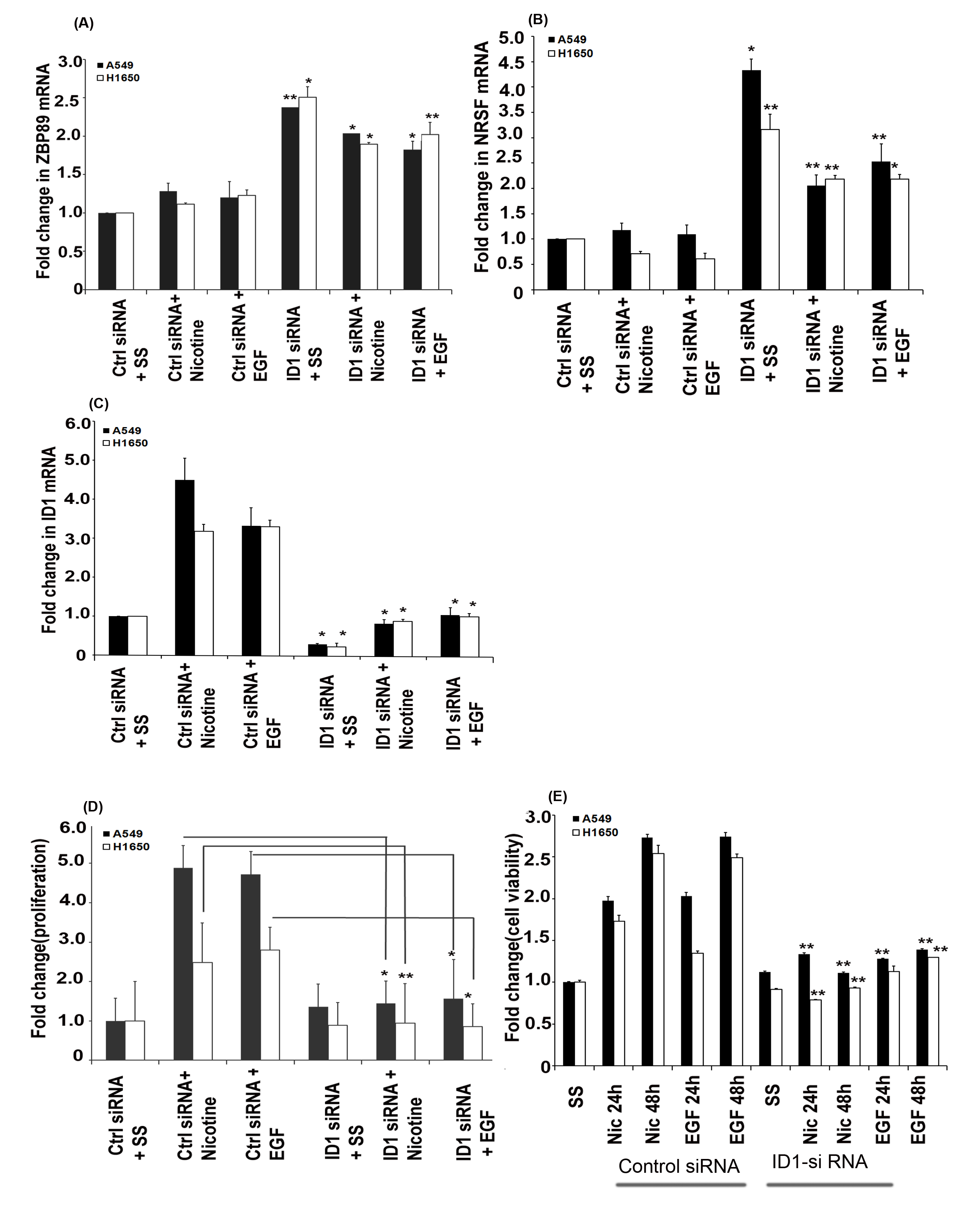


**Supplementry Figure S4: Depletion of ID1 by transient transfection up regulates ZBP89 & NRSF, whereas it abrogates the cell growth & proliferation in A549 & H1650** (A) RT-PCR showing upregulation of ZBP89 in the cells depleted of ID1 as compared to the non-targeting siRNA (B) RT-PCR showing upregulation of NRSF in the cells depleted of ID1 as compared to the non-targeting siRNA (C) RT-PCR confirming the down regulation of ID1 by transient siRNA transfection (D) Depletion of ID1 in A549 and H1650 cells significantly reduces the nicotine & EGF induced cell proliferation as seen in BrdU incorporation (E) Depletion of ID1 in A549 and H1650 cells significantly reduces the nicotine & EGF induced cell proliferation as seen by MTT assay. * represents *p* value <0.05 and ** represents *p* value < 0.0005.
